# Loss of the Two-Component System TctD-TctE in *Pseudomonas aeruginosa* Affects Biofilm Formation and Aminoglycoside Susceptibility in Response to Citric Acid

**DOI:** 10.1128/mSphere.00102-19

**Published:** 2019-03-06

**Authors:** Patrick K. Taylor, Li Zhang, Thien-Fah Mah

**Affiliations:** a Department of Biochemistry, Microbiology and Immunology, University of Ottawa, Ottawa, Ontario, Canada; University of Rochester

**Keywords:** *Pseudomonas aeruginosa*, biofilms, citric acid, two-component regulatory systems

## Abstract

The two-component system TctD-TctE is important for regulating the uptake of tricarboxylic acids in Pseudomonas aeruginosa. TctD-TctE accomplishes this through derepression of the gene *opdH*, which encodes a tricarboxylic acid-specific porin. Previous work from our lab revealed that TctD-TctE in P. aeruginosa also has a role in resistance to aminoglycoside antibiotics. The aim of this study was to further characterize the role of TctD-TctE in P. aeruginosa in the presence of citric acid. Here it was found that deletion of P. aeruginosa PA14 TctD-TctE (Δ*tctED*) resulted in a 4-fold decrease in the biofilm bactericidal concentrations of the aminoglycosides tobramycin and gentamicin when citric acid was present in nutrient media. Tobramycin accumulation assays demonstrated that deletion of TctD-TctE resulted in an increase in the amount of tobramycin retained in biofilm cells. The PA14 wild type responded to increasing concentrations of citric acid by producing less biofilm. In contrast, the amount of Δ*tctED* mutant biofilm formation remained constant or enhanced. Furthermore, the Δ*tctED* strain was incapable of growing on citric acid as a sole carbon source and was highly reduced in its ability to grow in the presence of citric acid even when an additional carbon source was available. Use of phenotypic and genetic microarrays found that this growth deficiency of the Δ*tctED* mutant is unique to citric acid and that multiple metabolic genes are dysregulated. This work demonstrates that TctD-TctE in P. aeruginosa has a role in biofilm development that is dependent on citric acid and that is separate from the previously characterized involvement in resistance to antibiotics.

**IMPORTANCE** Nutrient availability is an important contributor to the ability of bacteria to establish successful infections in a host. Pseudomonas aeruginosa is an opportunistic pathogen in humans causing infections that are difficult to treat. In part, its success is attributable to a high degree of metabolic versatility. P. aeruginosa is able to sense and respond to varied and limited nutrient stress in the host environment. Two-component systems are important sensors-regulators of cellular responses to environmental stresses, such as those encountered in the host. This work demonstrates that the response by the two-component system TctD-TctE to the presence of citric acid has a role in biofilm formation, aminoglycoside susceptibility, and growth in P. aeruginosa.

## Abstract (precis)

Nutrient availability is an important contributor to the ability of bacteria to establish successful infections in a host. Pseudomonas aeruginosa is an opportunistic pathogen in humans causing infections that are difficult to treat. In part, its success is attributable to a high degree of metabolic versatility. P. aeruginosa is able to sense and respond to varied and limited nutrient stress in the host environment. Two-component systems are important sensors-regulators of cellular responses to environmental stresses, such as those encountered in the host. This work demonstrates that the response by the two-component system TctD-TctE to the presence of citric acid has a role in biofilm formation, aminoglycoside susceptibility, and growth in P. aeruginosa.

## INTRODUCTION

Pseudomonas aeruginosa is a member of the gammaproteobacteria and an opportunistic pathogen in humans. It is a highly adaptable species and is readily able to sense and respond to its environment. In part, this is due to its genome encoding a large number of two-component systems (TCSs) that it uses to adapt to its surroundings (1, 2). This adaptability contributes to the ability of P. aeruginosa to colonize a variety of host environments (3, 4). Previous work has found that numerous TCSs in P. aeruginosa have a role in regulating virulence and the development of antibiotic resistance (5–9). TCSs are an extensively utilized means for bacteria to sense and respond to their environment (10–14). The basic makeup of a TCS consists of a membrane-bound sensor kinase and a cytoplasmic response regulator. Activation of the sensor kinase occurs through autophosphorylation of a histidine residue (14–16). The active kinase then phosphorylates its cognate response regulator, which activates it, and the active response regulator exerts its downstream transcriptional effects on gene expression (14–16). TCSs can potentially be involved in a multitude of regulatory pathways having diverse roles for cell responses (10, 17–20), including the development of antibiotic resistance (5, 8, 9, 21).

Salmonella enterica serovar Typhimurium, another pathogenic member of the gammaproteobacteria, contains an operon that encodes genes, named the tricarboxylic transporters (*tct*), which are responsible for the uptake of tricarboxylic carbon sources, such as citric acid (22). Within this operon are two genes for a TCS: *tctD*, which encodes a transcriptional regulator, and *tctE*, which encodes a histidine sensor kinase (22, 23). TctD-TctE senses tricarboxylic compounds in the environment and regulates the expression of the *tct* operon for uptake and metabolism of these compounds.

P. aeruginosa does not possess a fully conserved *tct* operon, but its genome does encode homologs of *tctD* and *tctE* (24). In P. aeruginosa, TctD-TctE acts on the expression of *opdH* through a mechanism of derepression in the presence of tricarboxylic acids. *opdH* encodes a porin (OpdH) required for the transport of tricarboxylic acids across the outer membrane permeability barrier of P. aeruginosa (24). Previous work from our lab found that a deletion of the P. aeruginosa operon *tctED* (previously described as PA0756-0757) in the PA14 wild-type strain resulted in a 4-fold increase in susceptibility to aminoglycoside antibiotics in biofilm cultures (25). Our current study questioned whether regulation of citrate metabolite (i.e., citric acid) uptake is involved in the aminoglycoside susceptibility phenotype observed in a *tctED* deletion (Δ*tctED*) strain.

In this study, we hypothesized that TctD-TctE is involved in biofilm formation as well as aminoglycoside resistance in P. aeruginosa. We observed that in biofilm cultures the Δ*tctED* mutant had a 4-fold increase in susceptibility to the aminoglycosides tobramycin and gentamicin when citric acid was present in the nutrient media. Furthermore, we found that there was a moderate but significant increase in the accumulation of tobramycin in Δ*tctED* mutant biofilms when citric acid was present in the growth medium. We further hypothesized that there are differences in biofilm formation in the Δ*tctED* mutant compared to PA14 in the presence of citric acid. We determined that in the presence of citric acid, the Δ*tctED* mutant showed no significant change in the amount of biofilm biomass, while the PA14 strain displayed reduced levels of biofilm biomass. It was also found that the Δ*tctED* mutant had a severely reduced level of growth when citric acid was present in the growth medium. The use of phenotypic microarrays determined that this phenotype was unique to citric acid as a carbon source. However, the use of a whole-genome expression microarray approach found that multiple metabolic genes not directly involved in citrate metabolism are dysregulated in the Δ*tctED* mutant relative to PA14. Here we demonstrate for the first time that TctD-TctE has a role in regulating biofilm formation in P. aeruginosa.

## RESULTS

### Loss of TctD-TctE leads to increased susceptibility to aminoglycosides in biofilm cultures.

Interest in studying TctD-TctE originated from previous findings from our lab demonstrating that the loss of expression of these proteins in the Δ*tctED* deletion strain (then designated the ΔPA0756-0757 strain) resulted in biofilms that were more susceptible to a subset of antibiotics, the aminoglycosides tobramycin and gentamicin (25). In this study, we wanted to explore whether inclusion of citric acid would have a further effect on the antibiotic susceptibility of the Δ*tctED* mutant. To explore this phenotype, we assayed for the minimal bactericidal concentrations (MBCs) of tobramycin and gentamicin with the addition of citric acid in the nutrient media (Table 1). We also included ciprofloxacin, a clinically relevant fluoroquinolone in the treatment of P. aeruginosa infections. Assays for determination of MBCs in planktonic cultures (MBC-P) containing 10 mM citric acid, in addition to antibiotic, found a 2-fold increase in susceptibility to both tobramycin and gentamicin in Δ*tctED* mutant cultures relative to PA14 cultures (Table 1). This increased susceptibility under planktonic conditions was not observed in our previous work, where citric acid was not included in the MBC assays (25). For assays for determination of MBCs in biofilms (MBC-B), a 4-fold increase in susceptibility to tobramycin and gentamicin was observed in the Δ*tctED* mutant compared to PA14 (Table 1). No difference in the MBCs between PA14 and the Δ*tctED* mutant was observed in either MBC-P or MBC-B assays with ciprofloxacin (Table 1). Additional antibiotics (including the antipseudomonal β-lactam ceftazidime, the fluoroquinolones levofloxacin and norfloxacin, chloramphenicol, and nalidixic acid) not previously tested (25) were assayed here. For all except one antibiotic, there were no differences in the MBC-Ps or MBC-Bs observed (see Table S1 in the supplemental material). A 2-fold increase in the MBC-P was observed when testing with the synthetic quinolone nalidixic acid.

**TABLE 1:** MBCs for antibiotics in medium including 10 mM citric acid

Culture and strain	MBC (μg/ml)		
	Tobramycin	Gentamicin	Ciprofloxacin
Planktonic			
PA14	64	128	4
Δ*tctED*	32	64	4
Biofilm			
PA14	400	800	40
Δ*tctED*	100	200	40

10.1128/mSphere.00102-19.5 TABLE S1 Minimal bactericidal concentrations (MBCs) for select antibiotics in media including 10 mM citric acid. Download Table S1, DOCX file, 0.02 MB. Copyright © 2019 Taylor et al. 2019 Taylor et al. This content is distributed under the terms of the Creative Commons Attribution 4.0 International license.

### The Δ*tctED* strain demonstrated increased accumulation of tobramycin in its biofilms.

**FIG 1:**
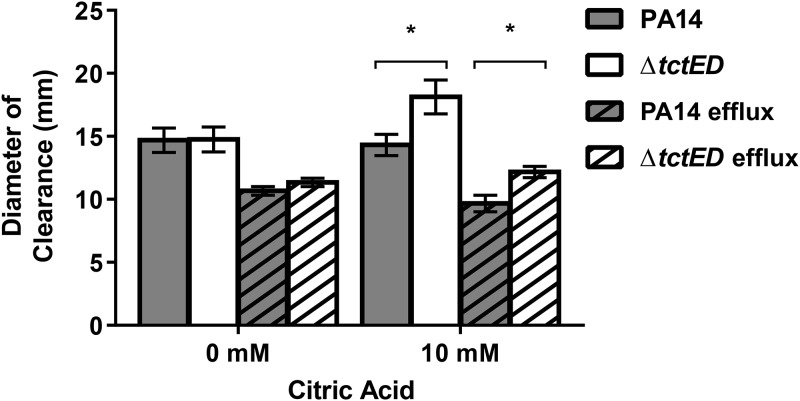
Tobramycin accumulation assays of PA14 and the Δ*tctED* mutant under conditions where citric acid is present during growth of the biofilms. By the disk diffusion assay, disks were loaded with whole-biofilm lysate from cultures grown under conditions where citric acid was present. The results represented by the columns are averages from 3 biological repeats, and error bars are SEMs. The Bonferroni-Dunn method was used to determine significance, *, *P* < 0.05.

To investigate this biofilm phenotype of tobramycin susceptibility, we questioned whether there was any greater accumulation of tobramycin in the Δ*tctED* mutant biofilms than in PA14 biofilms. We performed accumulation assays by growing biofilm cultures in the presence of citric acid (10 mM added to M63-arginine medium). The biofilms were then treated with a bactericidal concentration of tobramycin for the Δ*tctED* mutant (200 μg/ml) (Table 1) (26, 27). We included citric acid in this assay for the role that it plays in the activity of TctD-TctE (24) and also for its effects on cellular growth (28, 29) and biofilm formation (30). Biofilm cells were lysed and then assayed for the presence of tobramycin by measuring the diameter of the clearance zone on Escherichia coli lawns. M63 medium without added citric acid did not show any difference in tobramycin accumulation between the PA14 and Δ*tctED* strains (Fig. 1). However, when citric acid was present, there was a statistically significant (*P* < 0.05) increase in the zone of clearance observed, indicating a greater accumulation of tobramycin in Δ*tctED* mutant biofilms (Fig. 1). It is of note that a zone diameter change of ≥3 mm can clinically result in grouping of the bacteria to a susceptible or resistant category in antimicrobial disk susceptibility testing (31).

Efflux of antibiotics is a major mechanism of resistance for aminoglycosides, including tobramycin, in P. aeruginosa. Therefore, we were interested in testing whether a reduced level of efflux was contributing to the increased accumulation in the Δ*tctED* mutant by expressing the PA1875-1877 drug efflux system previously characterized by our lab (27) in both the PA14 and Δ*tctED* strains (producing the PA14^efflux^ and Δ*tctED*^efflux^ strains). We reasoned that these strains would determine if heightened efflux could restore wild-type levels of tobramycin accumulation. We found that there was a moderate reduction of the clearance zone on E. coli lawns for both the PA14^efflux^ and Δ*tctED*^efflux^ strains compared to that for the parental strains that did not express the efflux system. However, the trend of greater accumulation in the Δ*tctED*^efflux^ strain than in PA14^efflux^ was still observed (Fig. 1).

### Biofilm formation by the Δ*tctED* mutant is dysregulated in the presence of citric acid.

After observing that Δ*tctED* mutant biofilms have an increased accumulation of tobramycin in the presence of citric acid, we questioned whether there were differences in biofilm formation between the PA14 and Δ*tctED* strains using a crystal violet assay and microscopy.

**FIG 2:**
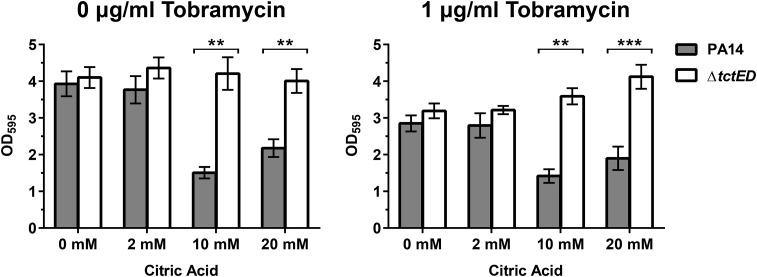
Amount of biofilm formation compared between PA14 and the Δ*tctED* mutant in the presence of added citric acid. Conditions of no tobramycin and tobramycin at a subinhibitory concentration (1 μg/ml) were tested. The results represented by the columns represent the means from 3 biological replicates, and error bars are SEMs. The Bonferroni-Dunn method was used to determine significance. **, *P* < 0.01; ***, *P* < 0.001.

In M63 medium with 23 mM arginine as a carbon source and no added citric acid, the PA14 and Δ*tctED* strains displayed similar levels of biofilm formation, as determined by staining with crystal violet (Fig. 2). With the introduction of citric acid into the nutrient media, Δ*tctED* mutant biofilms maintained similar levels of staining regardless of the concentration of citric acid present in each growth medium (Fig. 2). Conversely, PA14 displayed an inverse relationship, where increasing concentrations of citric acid resulted in smaller amounts of biofilm formation. This observation was most apparent at the highest concentration of 20 mM citric acid. The decreased level of biofilm formation in PA14 suggests that biofilm formation is altered in the Δ*tctED* mutant. These experiments were repeated with subinhibitory levels of tobramycin to determine if there were any greater effects on the observed biofilm; however, the trends were the same as those found when no tobramycin was added (Fig. 2).

Flagella and type IV pili are important for early steps in biofilm formation in P. aeruginosa (32, 33). These are also crucial components for swimming as well as swarming motility. Since biofilm formation in the Δ*tctED* mutant strain was altered, we assayed the swarming and swimming motility phenotypes of the PA14 and Δ*tctED* strains. We observed no difference between the two strains, suggesting that the defect in biofilm formation occurs at later stages of biofilm formation (Fig. S1).

10.1128/mSphere.00102-19.1 FIG S1 Swimming motility (A) and swarming motility (B) were no different in the Δ*tctED* strain relative to PA14. Plates were incubated for 18 h before images were captured. Images used are representative of those from 3 biological replicates. Swimming was performed on LB with 0.1% (wt/vol) agar. Swarming was performed as previously described by J. Overhage, M. Bains, M. D. Brazas, and R. E. W. Hancock (J Bacteriol **190:**2671–2679, 2008). Download FIG S1, EPS file, 1.4 MB. Copyright © 2019 Taylor et al. 2019 Taylor et al. This content is distributed under the terms of the Creative Commons Attribution 4.0 International license.

**FIG 3:**
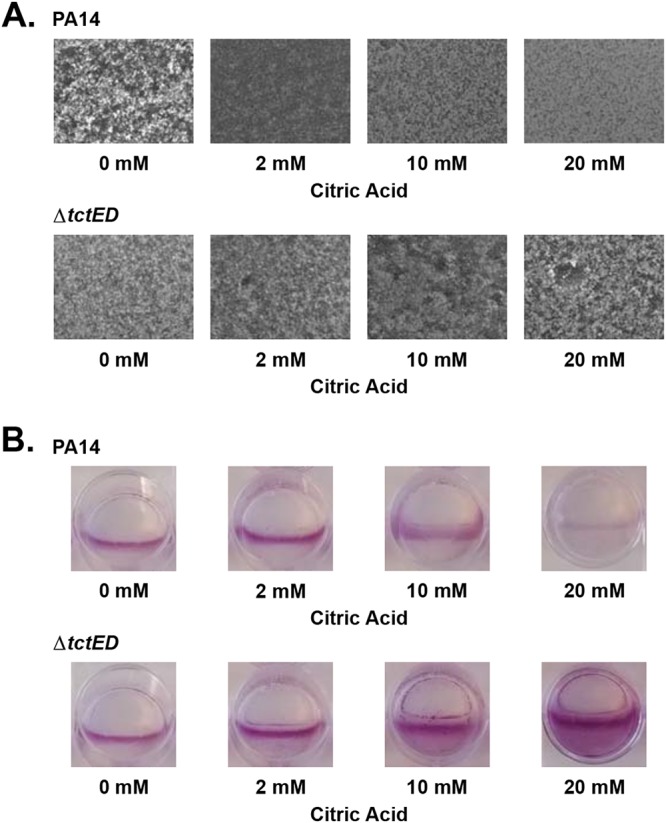
Phase-contrast microscopy (A) and 0.1% crystal violet staining (B) were used to assess PA14 and Δ*tctED* mutant biofilm cultures. Biofilms were grown statically in 12-well culture plates using the air-liquid interface assay described in Materials and Methods. Images are representative of those from 30 fields of view for each of 3 biological replicates. Crystal violet staining was performed on the same cultures used for microscopy after images were taken.

To further characterize the biofilm phenotype, we visualized PA14 and Δ*tctED* mutant biofilms by microscopy. Under conditions where no citric acid was added, PA14 biofilms were thick with macrocolonies present and covered the entire visual field. As increasing amounts of citric acid were added, the biofilms were flat and the surface was more sparsely populated in the presence of citric acid (Fig. 3A). To augment our analysis, we stained the biofilms that were formed in 12-well microtiter dishes with crystal violet. We observed that PA14 biofilm cultures decreased with increasing concentrations of citric acid (Fig. 3B). In contrast, the Δ*tctED* mutant maintained a high degree of biofilm formation in the presence of citric acid (Fig. 3A and B). In all concentrations of citric acid tested, the Δ*tctED* mutant maintained thick biofilms with visible dense macrocolonies (Fig. 3A). This biofilm growth appeared to increase with higher levels of citric acid, as observed by crystal violet staining (Fig. 3B). Typically, P. aeruginosa biofilms grow most densely at the air-liquid interface, observed as a line of crystal violet staining (Fig. 3B). This growth at the air-liquid interface was observed consistently in PA14. The Δ*tctED* mutant demonstrated higher levels of growth down beyond the air-liquid interface into the medium as the concentrations of citric acid increased (Fig. 3B). Taken together, these data suggest that the loss of *tctED* results in an inability to regulate biofilm formation in the presence of citric acid.

Addition of citric acid to our growth medium likely altered the pH. Therefore, we questioned whether the growth deficiency of the Δ*tctED* mutant was at all a result of changes in the pH of the medium. We measured the pH of M63 medium containing different concentrations of citric acid (0, 2, 10, and 20 mM), and the pH of M63 medium was adjusted accordingly (7.0, 6.8, 6.2, and 5.1, respectively). The growth of the Δ*tctED* strain showed that it displayed no difference from PA14 in its ability to grow in any of the pH-adjusted media (Fig. S2).

10.1128/mSphere.00102-19.2 FIG S2 Growth assays confirmed that the Δ*tctED* strain (Δ) displayed no difference in ability to grow relative to PA14 (●) in varying pH. Cultures were grown for 16 h with shaking, taking measurements every 30 min. Data shown are the means for 3 biological replicates, and error bars represent the standard error of the mean (SEM). For points that have no error bar displayed, the SEM was too small to be visible on the graph. The absorbance at 600 nm (OD_600_) was measured and converted to the number of CFU per milliliter of volume based on measuring OD_600_ and plating cultures of PA14 and the Δ*tctED* mutant in a separate assay. Download FIG S2, EPS file, 0.2 MB. Copyright © 2019 Taylor et al. 2019 Taylor et al. This content is distributed under the terms of the Creative Commons Attribution 4.0 International license.

### The Δ*tctED* strain has a growth deficiency in the presence of citric acid.

**FIG 4:**
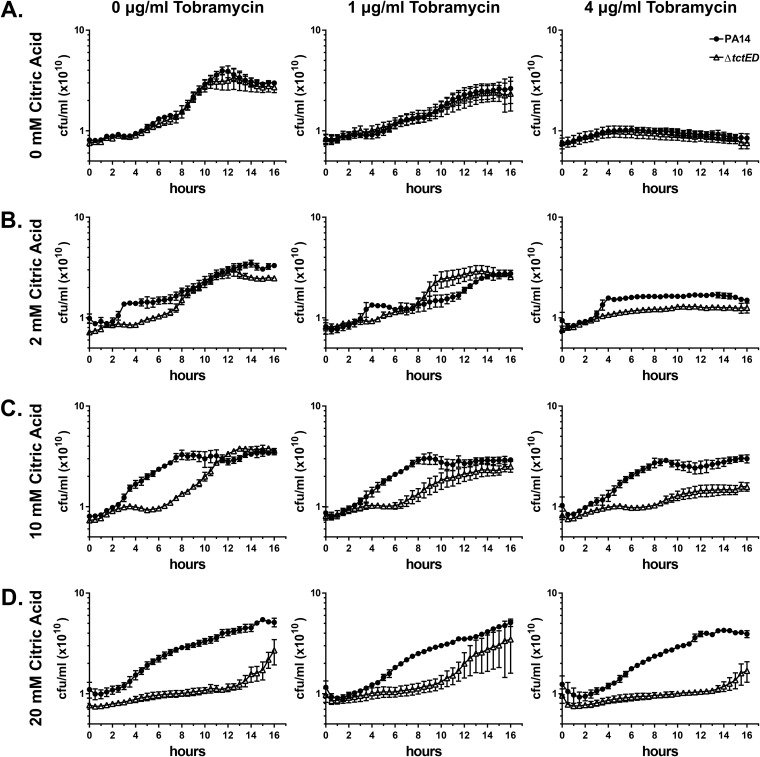
The Δ*tctED* strain (Δ) exhibits a reduced ability to grow relative to PA14 (●) when citric acid is present in M63-arginine medium. Concentrations of 0 mM (A), 2 mM (B), 10 mM (C), and 20 mM (D) citric acid were tested with zero, subinhibitory (1 μg/ml), and inhibitory (4 μg/ml) concentrations of tobramycin. The data shown are the averages from 3 biological replicates, where error bars are the standard error of the mean (SEM). Where no error bars are visible, the SEM was less than what could be displayed on the graph.

Since the Δ*tctED* strain displayed such a divergent biofilm phenotype in the presence of citric acid, we questioned whether there were any further growth phenotypes in citric acid. Previous research has found that high ratios of citrate metabolites relative to other carbon sources in nutrient media have an inhibitive effect on P. aeruginosa growth (28, 29). To test Δ*tctED* mutant growth in the presence of citric acid, we performed growth assays using M63 minimal medium containing arginine and various concentrations of both citric acid and tobramycin. When grown in higher concentrations of citric acid, reduced growth of the Δ*tctED* mutant relative to that of PA14 was observed (Fig. 4). In low and moderate concentrations (2 mM and 10 mM) of citric acid, there was a lag in growth of the Δ*tctED* strain, but it was able to achieve wild-type levels of culture densities by 16 h (Fig. 4B and C). The most extreme difference was observed at the highest concentration of citrate tested (20 mM), where the Δ*tctED* strain was severely reduced in its ability to grow compared to PA14 (Fig. 4D).

Given that the Δ*tctED* strain displayed an aminoglycoside-specific susceptibility, it was of interest to determine if challenge of the cultures with tobramycin would exacerbate this Δ*tctED* mutant growth deficiency in the presence of citric acid. For growth assays with tobramycin, subinhibitory (1 μg/ml) and inhibitory (4 μg/ml) concentrations were used (25). Regardless of the concentrations of tobramycin present, the Δ*tctED* mutant had the same observable trend of reduced growth relative to that of PA14 (Fig. 4).

**FIG 5:**
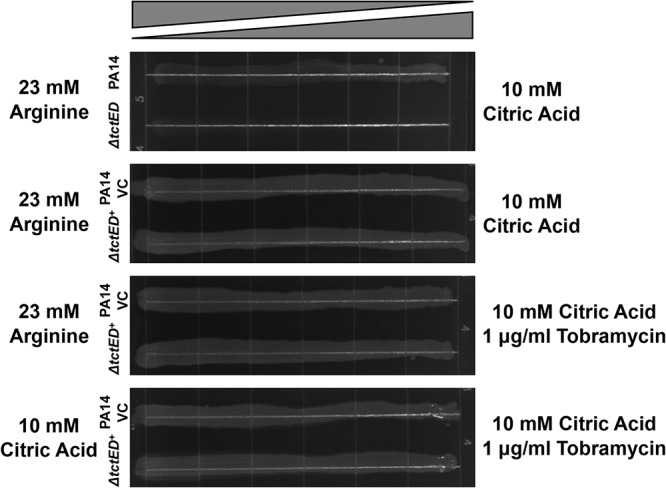
Complementation of the Δ*tctED* mutant with pJB866 harboring a wild-type copy of *tctED* (*tctED^+^*) restored growth on gradient agar plates. PA14 was transformed with pJB866 (PA14 VC [vector control]) to allow growth on selective medium.

To confirm that the citric acid growth deficiency observed in the Δ*tctED* mutant was due to the loss of TctD-TctE, a complementation strain (the *tctED^+^* strain) was struck out on gradient agar plates containing medium that had a continuous gradient of increasing citric acid concentrations in one direction. We were able to determine that expression of TctD-TctE from a pJB866 plasmid restored the ability of the strain to grow on citric acid as a sole carbon source (Fig. 5). Additionally, complementation with *tctED* also restored wild-type levels of resistance to tobramycin (Fig. 5).

### The Δ*tctED* mutant growth deficiency is unique to citric acid as a carbon source.

To determine if the Δ*tctED* strain had a growth deficiency on different carbon nutrient sources, we utilized high-throughput phenotypic microarrays containing a variety of carbon sources (34). These arrays provide a measure of changes in absorbance from reduction of a dye occurring in the medium, which indirectly provides a measure for aerobic respiration and cell growth. These arrays can then provide insight into the ability of a bacterial strain to metabolize specific nutrients. The Δ*tctED* strain displayed a moderately reduced ability to grow on the carbon sources α-hydroxy-butyric acid (plate and well position, PM1 and E7, respectively) and d,l-carnitine (PM2A and H5) relative to PA14 (Table 2 and Fig. S3). Additionally, there was a moderate increase in the growth of the Δ*tctED* strain relative to that of PA14 on adenosine (PM1 and E12), α-keto-butyric acid (PM1 and D7), putrescine (PM2A and H8), and uridine (PM1 and D12) (Table 2 and Fig. S3). Most notably, the Δ*tctED* mutant was unable to grow on citric acid as the sole carbon source (PM1 and F2) (Fig. S3). This growth deficiency was confirmed with independent growth assays where either 2.2 mM glucose or 5 mM, 10 mM, or 20 mM citric acid was provided as the sole carbon source for the PA14 and Δ*tctED* strains. Under conditions where citric acid was the only carbon source, Δ*tctED* had no observable growth, while PA14 grew under all conditions (Fig. S4).

**TABLE 2:** Assessment of Δ*tctED* mutant growth on various carbon sources through use of a phenotype microtiter assay

Carbon source	Δ*tctED* mutant growth relative to PA14
α-Hydroxy-butyric acid	−
α-Keto-butyric acid	+
Adenosine	+
Citric acid	−
d,l-Carnitine	−
Putrescine	+
Uridine	+

10.1128/mSphere.00102-19.3 FIG S3 Measures of oxidative phosphorylation performed on phenotypic microarray plates testing diverse carbon sources. PA14 (A) and Δ*tctED* (B) cells were grown for 24 h for these assays. Each curve represents increasing absorbance due to accumulation of the reduced form of a dye due to oxidative phosphorylation and is therefore an indirect measure of cell growth. In a single well of plates PM1 and PM2A, there is a unique carbon source available. Each plate is representative of 3 biological replicates. For each plate, well A1 contains no carbon source and is a negative control. Carbon sources present on the plate are available from the Biolog website (https://biolog.com/products-portfolio-overview/phenotype-microarrays-for-microbial-cells/). Download FIG S3, EPS file, 0.3 MB. Copyright © 2019 Taylor et al. 2019 Taylor et al. This content is distributed under the terms of the Creative Commons Attribution 4.0 International license.

10.1128/mSphere.00102-19.4 FIG S4 Growth assays measuring OD_600_ confirmed that the Δ*tctED* strain (Δ) could not use citric acid as a sole carbon source. The PA14 strain (●) was able to grow under all conditions tested. The carbon sources provided were 2.2 mM glucose (A) and 5 mM (B), 10 mM (C), and 20 mM (D) citric acid. Cultures were grown for 16 h with shaking, taking measurements every 30 min. The data shown are the means for 3 biological replicates, and error bars represent the standard error of the mean (SEM). For points that have no error bar displayed, the SEM was too small to be visible on the graph. Culture populations (number of CFU per milliliter) were calculated from the OD_600_ in a separate assay. Download FIG S4, EPS file, 0.1 MB. Copyright © 2019 Taylor et al. 2019 Taylor et al. This content is distributed under the terms of the Creative Commons Attribution 4.0 International license.

### Multiple genes that are involved in metabolism are dysregulated in the Δ*tctED* mutant.

DNA microarrays were used to better understand what pathways were dysregulated in the Δ*tctED* strain and possibly contributed to the observed growth deficiency and dysregulation of biofilm formation. It was found that under planktonic growth conditions 94 genes were dysregulated (>2-fold) and that under biofilm conditions 23 were significantly dysregulated (>2-fold) in the Δ*tctED* strain relative to the PA14 strain (Table 3). Of the 94 genes significantly dysregulated in planktonic cultures, 15 have roles in the metabolism in P. aeruginosa. In biofilm cultures, it was found that 8 of the 23 significantly dysregulated genes have a role in metabolic processes. Genes involved in metabolism that are significantly dysregulated are indicated by bold font in Table 3. Between planktonic and biofilm cultures, there was no overlap of the genes involved in metabolism, suggesting unique regulomes of TctD-TctE in each of these modes of growth. It was also notable that the transcriptional regulator genes *cheY*, *pmrA*, and *dnr*, as well as the two-component sensor gene *phoQ*, were significantly dysregulated in the Δ*tctED* strain between both the planktonic and biofilm conditions tested (Table 3).

**TABLE 3:** Genes with significant changes in expression (>2-fold) in the Δ*tctED* mutant relative to PA14 grown in planktonic and biofilm cultures

Culture and gene locus	Gene name^*a*^	Gene function	Fold change in expression^*b*^
Planktonic			
PA14_01310			4.80
PA14_70010			4.73
**PA14_01320**^*c*^	***coIII***	**Cytochrome *c* oxidase, subunit III**	**4.51**
PA14_60190	*clpB*	ClpB protein	3.81
PA14_07660			3.77
**PA14_49130**	***dctA***	**C_4_-Dicarboxylate transport protein**	**3.63**
**PA14_70040**	***dadA***	****d**-Amino acid dehydrogenase, small subunit**	3.06
PA14_38840			2.88
PA14_17550			2.82
PA14_07680			2.78
PA14_68840			2.75
PA14_58690			2.68
PA14_61600			2.68
PA14_61610			2.60
PA14_44480			2.57
PA14_02520			2.54
PA14_45620	*cheY*	Two-component response regulator CheY	2.48
PA14_17700			2.48
PA14_10730			2.44
PA14_24770			2.38
PA14_68260			2.36
PA14_45610	*cheZ*	Chemotaxis protein CheZ	2.29
PA14_16260			2.29
**PA14_15030**	***leuA***	**2-Isopropylmalate synthase**	**2.28**
PA14_61840			2.28
PA14_60520			2.26
PA14_16680			2.19
PA14_30830			2.17
PA14_44860			2.15
PA14_71720			2.14
**PA14_19870**	***ldh***	**Leucine dehydrogenase**	**2.08**
PA14_05060			2.06
PA14_24760			2.05
PA14_26590			2.04
PA14_51850			2.01
PA14_27210	*efp*	Translation elongation factor P	−2.01
PA14_66140			−2.01
**PA14_17080**	***pyrH***	**Uridylate kinase**	**−2.02**
PA14_64560			−2.02
PA14_65605	*parC*	Topoisomerase IV subunit A	−2.03
PA14_54370	*lepA*	GTP-binding protein LepA	−2.04
PA14_25490			−2.04
PA14_18350			−2.04
**PA14_61770**	***prs***	**Ribose-phosphate pyrophosphokinase**	**−2.06**
PA14_61780			−2.06
PA14_17060	*rpsB*	30S ribosomal protein S2	−2.09
**PA14_25740**	***tmk***	**Thymidylate kinase**	**−2.10**
PA14_51840			−2.12
**PA14_62710**	***pnp***	**Polyribonucleotide nucleotidyltransferase**	**−2.12**
PA14_21760	*capB*	Cold acclimation protein B	−2.13
PA14_65160			−2.14
**PA14_14440**	***valS***	**Valyl-tRNA synthetase**	**−2.15**
PA14_09030	*rpmD*	50S ribosomal protein L30	−2.15
**PA14_66230**	***waaG***	**UDP-glucose:(heptosyl) LPS alpha 1,3-glucosyltransferase**	**−2.15**
**PA14_17675**	***dgkA***	**Diacylglycerol kinase**	**−2.16**
PA14_09050	*secY*	Secretion protein SecY	−2.18
PA14_70640	*rubA1*	Rubredoxin 1	−2.20
PA14_73420	*rnpA*	Ribonuclease P protein component	−2.20
PA14_25760	*holB*	DNA polymerase III, delta prime subunit	−2.21
PA14_57580	*rpsI*	30S ribosomal protein S9	−2.22
PA14_09020	*rpsE*	30S ribosomal protein S5	−2.23
PA14_63120			−2.24
PA14_25730			−2.25
PA14_65170	*rpsR*	30S ribosomal protein S18	−2.25
PA14_58130	*mreC*	Rod shape-determining protein MreC	−2.26
PA14_41250	*tig*	Trigger factor	−2.27
PA14_08860	*rplD*	50S ribosomal protein L4	−2.30
PA14_12550			−2.33
PA14_09000	*rplF*	50S ribosomal protein L6	−2.36
**PA14_17220**	***lpxB***	**Lipid A-disaccharide synthase**	**−2.40**
PA14_25630	*rpmF*	50S ribosomal protein L32	−2.41
PA14_09010	*rplR*	50S ribosomal protein L18	−2.44
PA14_00580			−2.48
PA14_07560	*rpsU*	30S ribosomal protein S21	−2.48
PA14_63110			−2.48
PA14_14610			−2.49
PA14_63150	*pmrA*	Two-component regulator system response regulator	−2.49
**PA14_62830**	***tpiA***	**Triosephosphate isomerase**	**−2.53**
PA14_61820			−2.55
PA14_09040	*rplO*	50S ribosomal protein L15	−2.58
PA14_65180	*rpsF*	30S ribosomal protein S6	−2.58
PA14_15980	*rimM*	16S rRNA processing protein	−2.59
PA14_73410			−2.62
PA14_58570			−2.68
PA14_25270	*aroP1*	Aromatic amino acid transport protein AroP1	−2.76
**PA14_44060**	***sdhC***	**Succinate dehydrogenase (C subunit)**	**−2.80**
PA14_58120	*mreD*	Rod shape-determining protein MreD	−2.91
PA14_67560	*typA*	Regulatory protein TypA	−2.93
PA14_52340			−3.04
PA14_27370			−3.52
PA14_15970	*rpsP*	30S ribosomal protein S16	−3.55
PA14_70180	*rpmG*	50S ribosomal protein L33	−3.59
PA14_15990	*trmD*	tRNA (guanine-N1)-methyltransferase	−3.60
PA14_39060			−4.14
Biofilm			
PA14_72260			5.54
PA14_02520			5.26
PA14_42860			3.51
PA14_46900			3.41
PA14_56540			2.94
PA14_55750			2.92
PA14_22350			2.52
PA14_56690			2.47
**PA14_44470**	***hemN***	**Oxygen-independent coproporphyrinogen III oxidase**	**2.46**
**PA14_60700**	***ccpR***	**Cytochrome *c*_551_ peroxidase precursor**	**2.44**
PA14_49170	*phoQ*	Two-component sensor PhoQ	2.11
**PA14_52800**	***acsA***	**Acetyl coenzyme A synthetase**	**2.08**
**PA14_06870**	***dnr***	**Transcriptional regulator Dnr**	**2.06**
**PA14_20890**	***rfaD***	**ADP-**l**-glycero-**d**-mannoheptose 6-epimerase**	**2.02**
PA14_18360			2.02
**PA14_09980**	***dkgB***	**2,5-Diketo-**d**-gluconate reductase B**	**−2.06**
PA14_47420			−2.13
PA14_55590			−2.19
**PA14_57960**	***ptsN***	**Nitrogen regulatory IIA protein**	**−2.21**
PA14_00990			−2.22
PA14_56980			−2.23
PA14_52070			−2.24
**PA14_10170**	***fepB***	**Ferrienterobactin-binding periplasmic protein precursor**	**−2.70**

a Where no gene name is provided, it is an uncharacterized gene with a conserved hypothetical gene product.

b Values are the means for 2 biological replicates.

c Boldface describes genes involved in metabolism.

## DISCUSSION

The aim of this study was to further characterize the role of the TCS TctD-TctE in P. aeruginosa. We were able to build on previous findings and further characterize the aminoglycoside susceptibility of the Δ*tctED* deletion strain in the presence of citric acid. In this work, we also made the novel finding of a growth deficiency and biofilm dysregulation in the presence of citric acid for the Δ*tctED* strain.

First, to further characterize previous observations of an increased susceptibility to the aminoglycosides tobramycin and gentamicin in the Δ*tctED* mutant, we performed assays for MBC-P and MBC-B with citric acid included in the nutrient media. We found that there was a 4-fold increase in Δ*tctED* mutant susceptibility to tobramycin and gentamicin in MBC-B assays, similar to what was observed previously (Table 1). Interestingly, there was also a 2-fold increase in Δ*tctED* mutant susceptibility to these antibiotics in MBC-P assays that included 10 mM citric acid. Previous findings revealed no difference in MBCs between PA14 and the Δ*tctED* mutant in the absence of citric acid (25). It is likely that this small increase in planktonic susceptibility observed in the Δ*tctED* mutant was due to the added stress of a growth deficiency in the presence of citric acid that we observed here (Fig. 4) and that is discussed below. It was also noted here that there was a 2-fold greater MBC-P of the quinolone antibiotic nalidixic acid (see Table S1 in the supplemental material). The Δ*tctED* mutant had an MBC-P of 1,024 μg/ml, whereas an MBC-P of 512 μg/ml was observed in PA14. These MBC-Ps are higher than those of other antibiotics, such as tobramycin, gentamicin, and ciprofloxacin (32, 64, and 4 μg/ml in the Δ*tctED* mutant, respectively). It is possible that the differences seen with nalidixic acid are attributable to the fact that there is already a high intrinsic resistance to nalidixic acid in P. aeruginosa.

Further investigation of aminoglycoside susceptibility in the Δ*tctED* mutant led us to determine that there was a moderate increase in the accumulation of tobramycin in Δ*tctED* mutant biofilms grown in the presence of 10 mM citric acid (Fig. 1). A major mechanism of P. aeruginosa resistance to aminoglycosides is through efflux of the compound (35–37). We expressed an efflux system that contributes to biofilm-specific aminoglycoside resistance (27) in the PA14 and Δ*tctED* strains. Expression of this efflux system reduced the zones of clearance in E. coli and thereby indicated reduced tobramycin accumulation in biofilms of all P. aeruginosa strains (Fig. 1). However, there was still a moderately greater accumulation in the Δ*tcED* mutant than in PA14 (Fig. 1). This result demonstrates that efflux does not contribute to the tobramycin susceptibility observed in Δ*tctED* mutant biofilms.

Because the aminoglycoside susceptibility in the Δ*tctED* mutant was biofilm specific, we decided to further investigate biofilm formation in the presence of citric acid. Our analysis indicated that the Δ*tctED* mutant is unable to respond appropriately to the presence of citric acid. When biofilm cultures were grown, PA14 responded to increasing concentrations of citric acid with a reduction in biofilm formation, whereas the Δ*tctED* strain continued to form thick biofilms regardless of the increasing concentrations of citric acid (Fig. 2 and 3).

As stated above, Δ*tctED* displayed a consistently high biofilm mass for all concentrations of citric acid present in the medium tested. However, there were decreases in planktonic Δ*tctED* mutant culture growth (Fig. 4) with increases in the concentration of citric acid in the medium. It was also observed that the Δ*tctED* mutant could not grow if citric acid was provided as the only carbon source (Fig. S3 and S4). Through the use of phenotypic assays which measure oxidation levels in the medium by oxidative phosphorylation, therefore indicating the metabolic activity of the cultures, it was found that the Δ*tctED* mutant was uniquely inactive in citric acid-containing medium and that no other carbon source gave such low levels of activity relative to that of PA14 (Table 2 and Fig. S3). Taken together, these data suggest that the loss of TctD-TctE in the Δ*tctED* mutant has an effect on planktonic and biofilm growth due to an inability to properly sense and respond to citric acid present in the environment. Further support for this comes from the observation that complementing the Δ*tctED* mutant with a plasmid expressing TctD-TctE restored the ability to grow on citric acid as the sole carbon source, as seen in gradient plants (Fig. 5). It is known that high relative concentrations of citrate metabolites can be inhibitive of growth in P. aeruginosa (28, 29). It is therefore likely that the loss of *tctED* results in the dysregulation of sensation and the response to citric acid in the medium, leading to an inhibition of growth.

Other modes of growth were otherwise unaffected in the Δ*tctED* mutant when grown on regular M63 medium lacking citric acid. Through the use of swarming and swimming assays, we determined that there was no difference in motility between the PA14 and Δ*tctED* strains (Fig. S1). Both flagella and pili, which are important for motility, also have roles in forming biofilm structures in P. aeruginosa (32, 33). The lack of any motility defect in the Δ*tctED* mutant adds support to the idea that the loss of the TCS results in a dysregulation of signaling in biofilm formation and growth.

The heightened accumulation of tobramycin in the Δ*tctED* mutant may also be explained by a lack of response to citric acid in the medium. With the Δ*tctED* mutant maintaining higher levels of adherent biofilms than PA14, the accumulation of tobramycin may be at least in part due to a larger amount of biofilm cells and extracellular matrix in the Δ*tctED* mutant. With the greater accumulation of tobramycin in the matrix, the Δ*tctED* mutant would then acquire increasing localized concentrations of tobramycin and increased killing. It has been found previously that small molecules, such as aminoglycosides, readily permeate into the extracellular matrix of biofilms (38–40). It is possible that the accumulation observed is at least in part due to more tobramycin being retained in the matrix and biofilm cells.

While the Δ*tctED* mutant biofilm cultures displayed higher MBC-Bs (Table 1) and greater accumulation of tobramycin in medium containing citric acid (Fig. 1), it should be noted that when planktonic cultures of the Δ*tctED* mutant were challenged with tobramycin, there was only a slight change in the susceptibility phenotype (Fig. 4). This fits with previous data (25) and findings obtained in this study (Table 1) that there are minimal differences in Δ*tctED* planktonic cultures and that the greatest increases in susceptibility relative to that of PA14 are observed in biofilm cultures. This suggests that TctD-TctE activity has greater involvement in P. aeruginosa when it is developing as a biofilm than when it is developing as a planktonic culture. In biofilms, there are subpopulations of cells with various levels of metabolic activity (3, 39, 41). Because of the localized higher density of cells, there can be limitations of nutrients in those locations. It is reasonable that systems such as TctD-TctE would be more heavily relied upon in biofilms.

It is likely that other factors contribute to the role that TctD-TctE has in aminoglycoside susceptibility, considering that it is a TCS ultimately regulating transcriptional responses. This was evident based on genome-wide expression data from this study, which found that a wide range of genes were dysregulated in the Δ*tctED* mutant. Additionally, phenotypic microarray data suggested that the role that TctD-TctE has in P. aeruginosa is complex, affecting multiple different regulatory pathways between citrate uptake. A large portion of genes (46 of 94 in planktonic cultures and 15 of 23 in biofilms) that were dysregulated in the Δ*tctED* mutant are of unknown function in P. aeruginosa (Table 3). Of the genes that do have characterized functional roles, a high number of genes that are involved in metabolism through electron transport, catabolism, anabolism, or transport were represented (15 of 48 in planktonic cultures and all those in biofilms). Additionally, other members of two-component systems were represented, such as *cheY*, *phoQ*, and *pmrA* (Table 3). Many genes for ribosomal subunits, DNA replication, and cell shape were represented, but these might have been dysregulated indirectly as a result of reduced growth in the Δ*tctED* mutant.

Phenotypic microarray analysis found that the Δ*tctED* mutant was completely defective in its ability to grow with citric acid as a sole carbon source. Interestingly, the Δ*tctED* mutant had moderately increased growth relative to PA14 in medium with carbon sources of α-keto-butyric acid, adenosine, putrescine, and uridine (Table 2) and moderately decreased growth in α-hydroxy-butyric acid and d,l-carnitine. It has recently been shown that the metabolic activity of bacteria alone can have a significant effect on susceptibility to antibiotic treatment (42, 43). It is possible that metabolic dysregulation in general also has a contribution to the aminoglycoside susceptibility observed in the Δ*tctED* mutant.

In conclusion, this study has further resolved some contributors to the role that TctD-TctE has in resistance and tolerance to aminoglycosides in P. aeruginosa through finding that the loss of TctD-TctE results in increased aminoglycoside susceptibility in the biofilm when citric acid is present in the environment. Furthermore, we were able to characterize the biofilm phenotype of consistent biofilm mass as well as a growth deficiency due to the loss of TctD-TctE in P. aeruginosa when citric acid is present. This work emphasizes the importance of how TCSs responsible for sensing and responding to environmental cues, even those for metabolites, can have significant impacts on biofilm development and when cells encounter dynamic and changing environmental conditions, which is important for developing improved methods of treating infections caused by bacterial pathogens.

## MATERIALS AND METHODS

### Bacterial strains and growth conditions.

The strains and plasmids used in this study are listed Table 4. All cultures except those specified were grown overnight at 37°C with constant shaking in LB from single colonies grown on LB agar plates. For the experiments, overnight cultures were subcultured into M63 minimal medium [22 mM KH_2_PO_4_, 40 mM K_2_HPO_4_, 15 mM (NH_4_)_2_SO_4_], using 0, 2, 10, and 20 mM citric acid and 23 mM arginine as carbon sources. For strains carrying pJB866 constructs, the medium was supplemented with 2 mM *m*-toluic acid for promoter induction and 10 μg/ml tetracycline for maintenance of plasmids. The only exception for the growth medium under experimental conditions was for growth assays conducted using Biolog plates on an OmniLog system, where the buffer and nutrient sources provided by the manufacturer of PM1 and PM2A plates were used (https://biolog.com/products-portfolio-overview/phenotype-microarrays-for-microbial-cells/).

**TABLE 4:** Strains and plasmids used in this study

Strain	Description	Reference or source
Strains		
PA14	P. aeruginosa wild-type strain	25
Δ*tctED*	PA14 containing a chromosomal deletion of *tctED*	25
PA14 VC	PA14 complemented with pJB866	25
* tctED^+^*	Δ*tctED* mutant complemented with pJB866::*tctED*	25
PA14^efflux^	PA14 complemented with pJB866::PA1875-1877	This study
Δ*tctED*^efflux^	Δ*tctED* mutant complemented with pJB866::PA1875-1877	This study
Plasmids		
pJB866	*Pm m-*toluic acid-inducible promoter; tetracycline resistant	44
pJB866::*tctED*	pJB866 carrying a *Pm*-regulated insertion of *tctED*	25
pJB866::PA1875-1877	pJB866 carrying PA1875-1877 drug efflux system genes	27

Biofilms grown for microscopy were cultured using an air-liquid interface assay described previously (25). Overnight cultures were subcultured 1/100 into fresh medium in 12-well plates. Cultures were incubated statically for 24 h at 37°C with the plates propped up at an angle of ∼30 to 45°. Before microscopy, the media with planktonic cultures were removed and the wells were washed once with sterile M63 buffer. A volume of 200 μl of fresh M63 buffer was added to the wells to prevent the biofilms from drying out while performing microscopy, and the culture plate was put directly on the microscope stage for analysis.

The absorbance, or the optical density at a 600-nm wavelength (OD_600_), was measured to determine culture growth. The number of CFU per milliliter of volume for growth curves was calculated by measuring the OD_600_ of separate PA14 and Δ*tctED* mutant cultures, plating out dilutions, and performing colony counts to determine the number of CFU per milliliter. The PA14 and Δ*tctED* strains displayed no difference in the relationship of the OD_600_ measurements to the plated colony counts. Based on the medium and conditions used in this study, an OD_600_ of 1.0 represents a culture population of 6 × 10^10^ CFU/ml for P. aeruginosa. This value was used to calculate all values of the number of CFU per milliliter from OD_600_ measurements.

### MBC assays.

Assays for determination of minimal bactericidal concentrations (MBCs) in biofilms (MBC-Bs) and in planktonic cultures (MBC-Ps) were utilized to assess susceptibility to antibiotics (25–27). An aliquot of overnight cultures was diluted (1/50) into fresh M63 medium with arginine as described above and then used for final inoculation of antibiotic-containing medium. For the MBC assays, a final concentration of 10 mM citric acid was added to the medium. To prepare MBC-B assay mixtures, diluted cultures were grown statically for 24 h at 37°C to form mature biofilms. After 24 h, the planktonic cultures and medium were removed and fresh medium containing antibiotics was added. Twofold serial dilutions of antibiotics ranged from 2.5 to 160 μg/ml for ciprofloxacin, 12.5 to 800 μg/ml for gentamicin, and 6.25 to 400 μg/ml for tobramycin. The preformed biofilms were incubated in the presence of antibiotics for 24 h at 37°C. For the MBC-P assays, diluted cultures were treated with antibiotics and incubated for 24 h at 37°C. Antibiotic concentration ranges were 0.5 to 32 μg/ml for ciprofloxacin, 2 to 128 μg/ml for gentamicin, and 1 to 64 μg/ml for tobramycin. After incubation, the cultures used for both MBC-B and MBC-P determination were spotted onto LB agar plates and grown overnight at 37°C. The concentration in the culture from which spots that displayed no growth upon subculture were sampled was taken as the MBC.

### Tobramycin accumulation assays.

Tobramycin accumulation assays were performed as previously described (27). Briefly, static biofilms of the PA14 and Δ*tctED* strains were grown in 12-well microtiter plates for 24 h with or without 10 mM citric acid supplemented in the medium. Biofilms were treated with 200 μg/ml tobramycin for 8 h and subsequently rinsed with M63 buffer. The biofilms were treated with 0.1 M glycine (pH 3) at 37°C to lyse the biofilm cells. After 16 h, the mixture of lysed cells and glycine buffer was evaporated to dryness, resuspended in sterile water, and absorbed into sterile Whatman paper disks. The disks were then placed on agar plates spread with E. coli DH5α. The plates were incubated at 37°C for 16 h, and the zone of clearing around the disk was used as an indication of the amount of tobramycin in the biofilm lysate.

### Crystal violet staining.

Assessment of biofilm mass was done by staining with 0.1% (wt/vol) crystal violet. Static biofilms in the respective media were grown for 24 h in 96-well plates. The cell population was first taken by reading the OD_600_ and converting the value to the number of CFU per milliliter. Planktonic cultures were then removed, and 100 μl of 0.1% crystal violet were added to the wells and allowed to stain for 20 min. The stain was then removed, the wells were washed, and the crystal violet absorbed by the biofilms was solubilized with 70% ethanol. The OD_595_ were then read as an assessment of the amount of biofilm mass adherent within the wells.

### Microscopy.

A Leica DMI6000 B inverted microscope with the companion Leica Application Suite software provided (v1.5.1, build 869) was used. Images are representative of those from 3 biological replicates, for each of which 30 fields of view were assessed.

### Phenotype microarrays testing various carbon sources.

Phenotypic assessment of growth for a collection of carbon sources was performed using Biolog MicroPlates. Overnight cultures were diluted to a McFarland standard of 0.25 in buffer with dye added; both the buffer and the dye were provided by Biolog, Inc. Plates PM1 and PM2A were utilized to assess the growth on various carbon sources. Assays were performed on a GEN III OmniLog ID system, where the plates were incubated for 24 h and readings were taken every 15 min. Three biological replicates were performed.

### Gradient agar plates.

Agar plates containing continual transitions of one nutrient medium to another were prepared as previously described (38). Culture plates were set at an angle as one nutrient medium was poured, allowed to set, and laid flat before the other nutrient medium was poured on top, generating a gradient of one medium to another. Overnight cultures of P. aeruginosa strains were then dragged along the gradient using an inoculating loop.

### RNA isolation.

RNA was isolated by lysis of whole cells grown under planktonic and biofilm conditions. Planktonic cultures were grown by subculturing overnight cultures into fresh M63 medium containing arginine as the sole carbon source. The cultures were then grown at 37°C with constant shaking for 4 to 5 h until reaching an OD_600_ approaching 0.5, before pelleting and lysing the cells. Biofilm cultures were grown by spotting 48 5-μl aliquots of an overnight culture onto M63-arginine agar plates. The plates were first grown for 24 h at 37°C and then grown for another 24 h at room temperature before harvesting the biofilm cultures. RNA was isolated from the cultures by first lysing the pelleted cultures using the TRIzol reagent. RNA was purified using a PureLink RNA minikit according to the instructions of Thermo Fisher Scientific, Inc. RNA samples were cleared of DNA through the use of DNase digestion, and samples were checked for DNA contamination by PCR.

### Microarray analysis.

Microarrays were performed using an Affymetrix GeneChip system for the P. aeruginosa PAO1 annotated genome. Samples were sent to the Genome Québec and Innovation Centre at McGill University, Montréal, QC, Canada, for quality control testing and performing the microarrays. Raw data were received as .cel files and analyzed in-house. Expression Console software, build 1.3.1.187, and the annotated P. aeruginosa PAO1 library Pae_G1a were acquired from the Affymetrix website. While PA14 background strains were used in this study, the close genetic homology of the core genome between P. aeruginosa strains made it sufficient to use PAO1 DNA microarray chips and the Pae_G1a library. Data normalization was performed using the robust multiarray average method, and the housekeeping gene *rpoD* was selected for expression normalization. Gene expression changes are represented as the mean fold change in expression in the Δ*tctED* mutant relative to that in PA14 under planktonic or biofilm conditions for 2 biological replicates of each condition.

### Accession number(s).

The data have been deposited in the Gene Expression Omnibus (GEO) database under accession number GSE114431.
