# Supplementary material for: Loss of the Two-Component System TctD-TctE in Pseudomonas aeruginosa Affects Biofilm Formation and Aminoglycoside Susceptibility in Response to Citric Acid
Source: mSphere. 2019 Mar 6;4(2):e00102-19. doi: 10.1128/mSphere.00102-19 (PMC6403454; doi:10.1128/mSphere.00102-19)
Supplement: TABLE S1 [file mSphere.00102-19-st001.docx]

**Table S1**

|  | **MBC Planktonic (μg/ml)** | | | | |
| --- | --- | --- | --- | --- | --- |
|  | **Ceftazidime** | **Chloramphenicol** | **Levofloxacin** | **Nalidixic Acid** | **Norfloxacin** |
| **PA14** | >1,024 | >2,048 | 2 | 512 | 8 |
| **Δ*tctED*** | >1,024 | >2,048 | 2 | 1,024 | 8 |
|  | **MBC Biofilm (μg/ml)** | | | | |
|  | **Ceftazidime** | **Chloramphenicol** | **Levofloxacin** | **Nalidixic Acid** | **Norfloxacin** |
| **PA14** | >6,400 | 3,200 | 25 | 3,200 | 400 |
| **Δ*tctED*** | >6,400 | 3,200 | 25 | 3,200 | 400 |
